# Some Advice to Those Now Entering

**Published:** 1920-09-04

**Authors:** 


### DENTISTRY AS A PROFESSION.

### Some Advice to those Now Entering.

At the present time few professions can offer greater chances of success and prosperity to a student than that of dental surgery. We have a profession at present only in its infancy, but which in due time will probably have as many licentiates upon its register as its sister profession of medicine. Within the last decade the importance of oral hygiene has been fully recognised by the medical faculty, and in a lesser degree by the public, and in the present year the newly instituted Ministry of Health is making great efforts to cope with the degenerate dentition of the nation. An administrative dental staff is being enlisted, and it is computed that at the present time all, or nearly all, the available registered dental surgeons might be absorbed in this work alone! But we are a nation of some forty-five millions, and so we have no hesitation whatever in recommending dentistry as a profession into which many thousands of students may be absorbed before there would be the slightest semblance of

**Dentistry as a Profession**—(*continued*).

overcrowding. Dental surgery occupies, in fact, a unique position among the professions, and is one which well deserves serious contemplation by any parent before launching his boy into a professional career.

### Preliminary Examinations.

Before a candidate can be entered on the roll of registered dental students, he must have attained the age of sixteen years, and have passed a recognised examination in general education, comprising subjects specified by the General Medical Council of Great Britain and Ireland.

The matriculation or graduate examinations of any English University are accepted, and in addition the senior local examinations of Oxford and Cambridge, and also a special examination instituted by the College of Preceptors for this purpose.

### Qualification.

The qualification which is most popular in England is that of the L.D.S.R.C.S. (Eng.); Licentiate in Dental Surgery of the Royal College of Surgeons of England.

In addition, University Degrees (B.D.S. and M.D.S., Bachelor and Masters of Dental Surgery) are granted by the Universities of Birmingham, Manchester, Liverpool, and Durham; London University has recently instituted an M.S. degree (Master of Surgery) which may be taken in Dental Surgery, but before this examination can be attempted the candidate must have graduated in both Medicine and Surgery (M.B., B.S. (Lond.) ) at this University.

The Royal College of Surgeons of Edinburgh have also this year decided to award a "Higher Dental Diploma" (H.D.D. (Edin.)), which is an attempt to institute an examination comparable to the Fellowship of the Royal College of Surgeons of Edinburgh. No degree in Dental Surgery is at present awarded by the older Universities of Oxford and Cambridge, but the matter is under the consideration of the Senates, and the installation of "chairs" in this subject will take place in the next year or so.

The most satisfactory qualification, as we have mentioned, is the L.D.S.R.C.S. (Eng.), and this is really all that is required by the average student; but if he is ambitious and wishes for a post on the staff of a teaching dental hospital, he must possess a medical qualification as a *sine quâ non.* The medical qualification usually taken is that of the Conjoint Board (M.R.C.S. (Eng.), L.R.C.P. (Lond.)).

The course of the dental student is only lengthened by about eighteen months to two years in taking this additional qualification, as so many of the subjects overlap in the two curricula.

### Dental Schools.

Dental schools exist in most of the large towns of Britain, and the largest are situated in the three capital cities of London, Edinburgh, and Dublin. In London there is a special hospital—the Royal Dental Hospital of London—and the dental departments of the London, University, and Guy's Hospitals, the last-named being the largest in the United Kingdom. If possible, the dental student should join a "general" rather than a "special" hospital, in order that both medical and dental studies may be carried out under one roof; much time and delay being saved in this way.

### Course of Study.

The first year is devoted to the study of science for the "Preliminary Science Examination," in which the student is examined in the elements of physics and chemistry; such study is usually undertaken at the hospital, but, if it is wished, the General Medical Council allow this preliminary work to be carried out at a public school or institution duly recognised by the examining authorities for that purpose.

The second year is given up to the study of dental mechanics and dental metallurgy, these subjects comprising what is known as the First Professional Examination. Dental mechanics are usually taught at the laboratories of the hospital, but, under a somewhat archaic by-law of the College of Surgeons students are still allowed to be "articled" or "apprenticed" to a registered dental surgeon for this purpose; but we strongly advise students to eschew such practice, and to pursue their study of these most important subjects under the auspices of their skilled hospital tutors.

Once the final examination is passed the candidate receives his diploma, but is not allowed to practise until he has registered as a dental surgeon at the offices of the General Medical Council.

### Post-Graduate Study.

The newly qualified practitioner is strongly advised to secure at least a year's further experience before starting for himself. The most ideal course of study would be to receive the appointment of dental house-surgeon at his own hospital; but such posts are limited and competitive, so that only the academically distinguished will be favoured with the priceless experience which such an appointment affords. Therefore, in the great majority of cases a post as an assistant or "locum tenens" to a registered dentist should be sought. Such posts are at present very well paid, and at least £10 per week may be earned in this way. Once experience has been gained, success is assured, because the field for new dental surgeons is a large one. There is no need to purchase a practice—indeed, there are very few for sale—as in a few months a fresh individual practice may be established in almost any district in the country, so great is the shortage of registered dentists. The status of the profession is continually improving, and within a few months fresh legislation will probably remove all unregistered practitioners. The whole profession, indeed, promises to be united and ethical, well able to take its place beside its sister in every sense of the word, and its outlook is decidedly good.

